# Effects of Harvest Time on Phytochemical Constituents and Biological Activities of *Panax ginseng* Berry Extracts

**DOI:** 10.3390/molecules24183343

**Published:** 2019-09-13

**Authors:** Seung-Yeap Song, Dae-Hun Park, Seong-Wook Seo, Kyung-Mok Park, Chun-Sik Bae, Hong-Seok Son, Hyung-Gyun Kim, Jung-Hee Lee, Goo Yoon, Jung-Hyun Shim, Eunok Im, Sang Hoon Rhee, In-Soo Yoon, Seung-Sik Cho

**Affiliations:** 1 Department of Pharmacy, College of Pharmacy, Mokpo National University, Jeonnam 58554, Korea; tgb1007@naver.com (S.-Y.S.); gyoon@mokpo.ac.kr (G.Y.); s10004jh@gmail.com (J.-H.S.); 2 Department of Nursing, Dongshin University, Jeonnam 58245, Korea; dhj1221@hanmail.net; 3 Department of Pharmacy, College of Pharmacy, Pusan National University, Busan 46241, Korea; sswook@pusan.ac.kr (S.-W.S.); eoim@pusan.ac.kr (E.I.); 4 Department of Pharmaceutical Engineering, Dongshin University, Jeonnam 58245, Korea; parkkm@dsu.ac.kr; 5 College of Veterinary Medicine, Chonnam National University, Gwangju 61186, Korea; csbae210@chonnam.ac.kr; 6 School of Korean Medicine, Dongshin University, Jeonnam 58245, Korea; hsson@dsu.ac.kr; 7 Department of Research Planning, Mokpo Marine Food-industry Research Center, Jeonnam 58621, Korea; khg8279@naver.com (H.-G.K.); bluebabyi@nate.com (J.-H.L.); 8 Department of Biological Sciences, Oakland University, Rochester, MI 48309, USA; srhee@oakland.edu

**Keywords:** ginseng berry, harvest time, ginsenoside, antioxidant activity, anti-elastase activity

## Abstract

Ginseng (*Panax ginseng*) has long been used as a traditional medicine for the prevention and treatment of various diseases. Generally, the harvest time and age of ginseng have been regarded as important factors determining the efficacy of ginseng. However, most studies have mainly focused on the root of ginseng, while studies on other parts of ginseng such as its berry have been relatively limited. Thus, the aim of this study iss to determine effects of harvest time on yields, phenolics/ginsenosides contents, and the antioxidant/anti-elastase activities of ethanol extracts of three- and four-year-old ginseng berry. In both three- and fourfour-year-old ginseng berry extracts, antioxidant and anti-elastase activities tended to increase as berries ripen from the first week to the last week of July. Liquid chromatography-tandem mass spectrometry analysis has revealed that contents of ginsenosides except Rg1 tend to be the highest in fourfour-year-old ginseng berries harvested in early July. These results indicate that biological activities and ginsenoside profiles of ginseng berry extracts depend on their age and harvest time in July, suggesting the importance of harvest time in the development of functional foods and medicinal products containing ginseng berry extracts. To the best of our knowledge, this is the first report on the influence of harvest time on the biological activity and ginsenoside contents of ginseng berry extracts.

## 1. Introduction

Ginseng (*Panax ginseng*) has long been used as a traditional medicine for the prevention and treatment of various diseases, including cancer, diabetes, inflammation, allergy, and cardiovascular diseases, in the East Asia, particularly in Korea and China [1,2]. Ginseng is one of the best known and most recognized medicinal herbs. Its pharmacological effects have been successfully demonstrated by numerous studies worldwide [3]. However, most studies have focused mainly on the root of ginseng, while studies on other parts of ginseng such as its berry and leaf are relatively limited [4].

More than sixty different ginsenosides have been identified from various parts of ginseng [2]. In particular, ginseng berry is known to have a distinct phytochemical profile. It contains significantly higher ginsenoside content than ginseng root [5,6]. Oral bioavailability of ginsenosides is generally very low. It is only 0.64% for Rb1 and 3.29% for Rg1 in rats [7,8]. However, oral absorption of ginsenoside Re is significantly higher (by 1.18–3.95 fold) after oral ingestion of a ginseng berry extract than pure ginsenoside Re [9]. To date, a few in vitro and in vivo studies have reported a variety of biological activities of ginseng berry on cancer, diabetes, sexual dysfunction, skin whitening, immunity, and liver injury. These studies are summarized in Table 1 [3,4,10,11,12,13,14,15,16]. A randomized and placebo-controlled clinical trial has also been performed to evaluate the safety and efficacy of ginseng berry extract on glycemic control [17].

A recent study reported the alterations of metabolomes during five different ginseng berry maturation stages and their effects on the functional bioactive compounds in ginseng [18]. Thus, information regarding the optimal harvest time of ginseng berry is needed to standardize the collection and pretreatment process of the plant material for its further development as functional foods or medicinal preparations. However, only a few studies have reported the influence of harvest time on the chemical and biological properties of ginseng berry. In a previous study, five different flower and berry development stages (flower bud, flowering, early berry, green berry, and red berry) were tested with respect to ginsenoside biosynthetic gene expression and ginsenoside contents in biochemical and molecular aspects [19]. However, we focused on the effect of harvest time on biological activities and chemical profiles of green-to-red ginseng berries, which is more relevant to the agricultural and industrial aspects. Here, the objective of the present study is to determine the effects of harvest time on yields, phenolic contents, ginsenoside contents, antioxidant activity, and the elastase inhibitory activity of ethanol extracts of three and four years old ginseng berry.

## 2. Results and Discussion

### 2.1. Drying and Extraction Yields of Ginseng Berry Extracts

To date, there have been no reports on yields related to the preparation of ginseng berry extracts. As shown in Table 2, after drying the harvested ginseng berry with hot air, the yield of this process ranged from 29.9% to 34.8% (31.8% on average). After extracting the dried ginseng berry with 70% ethanol, the yield of this process ranged from 8.8% to 12.6% (mean: 10.8%). The overall production yield of the extract was calculated to be 3.4%.

### 2.2. Antioxidant Properties of Ginseng Berry Extracts

**Figure 1:**
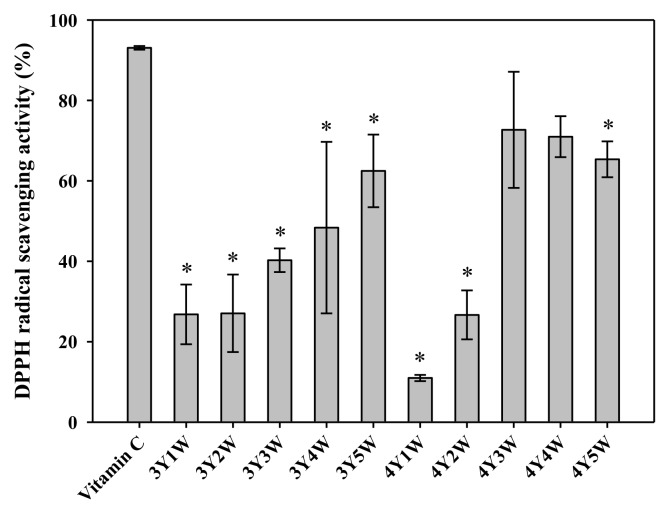
DPPH radical scavenging activity of ginseng berry extracts harvested at various time points. Rectangular bars and their error bars represent means and standard deviations, respectively (*n* = 3). The ‘*m*Y*n*W’ on the x-axis means *m*-year-old ginseng berry harvested in the *n*th week of July. *, significantly lower than the ‘Vitamin C’ group (positive control).

**Figure 2:**
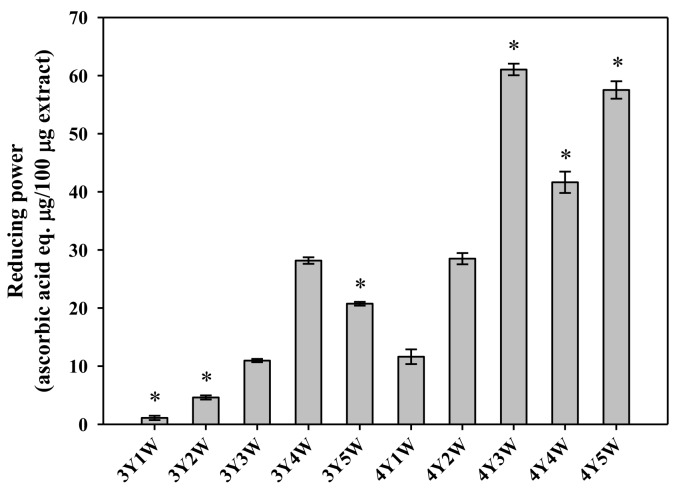
Reducing power of ginseng berry extracts harvested at various time points. Rectangular bars and their error bars represent means and standard deviations, respectively (*n* = 3). The ‘*m*Y*n*W’ on the x-axis means *m*-year-old ginseng berry harvested in the *n*th week of July. *, significantly different from other groups.

**Figure 3:**
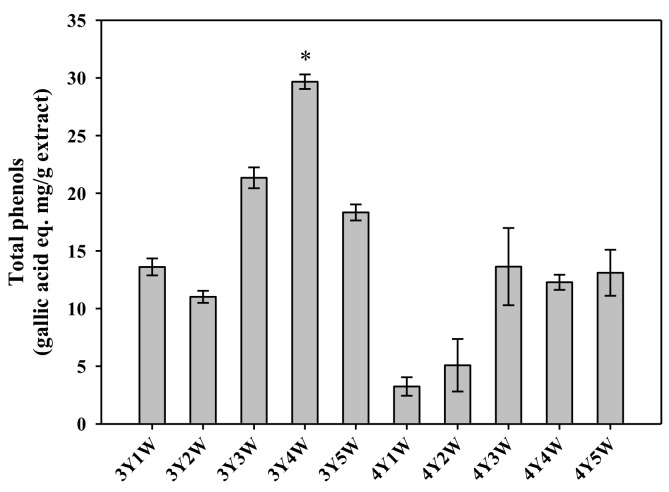
Total phenolic contents of ginseng berry extracts harvested at various time points. Rectangular bars and their error bars represent means and standard deviations, respectively (*n* = 3). The ‘*m*Y*n*W’ on the x-axis means *m*-year-old ginseng berry harvested in the *n*th week of July. *, significantly different from other groups.

Antioxidant properties of ginseng berry extracts were assessed by measuring DPPH radical scavenging activity, reducing power, and total phenolic contents. DPPH antioxidant assay is a fast and easy method to evaluate free radical scavenging capacity of a given sample [20]. As shown in Figure 1, DPPH radical scavenging activities of three-year-old ginseng berry extracts tended to increase from 26.8% to 62.5% when the harvest time was delayed. Those of four-year-old ginseng berry extracts also showed similar tendency of increase from 11.0% to 72.7%. Extracts of four-year old ginseng berry harvested in the 3rd and 4th weeks of July exhibited DPPH radical scavenging activity comparable to the positive control (vitamin C), which tended to be higher than other groups (Figure 1). As shown in Figure 2, the reducing power tended to increase as the harvest time was delayed from the 3rd year 1st week (3Y1W) to 4th year 5th week (4Y5W). Extracts of four-year-old ginseng berry harvested in the 3rd week of July exhibited significantly higher reducing power than other groups (Figure 2). As shown in Figure 3, total phenol contents of three-year-old ginseng berry extracts tended to increase from 13.6% to 29.7% as the harvest time was delayed from 1st week to 5th week of July. Those of four-year-old ginseng berry extracts showed a similar tendency, increasing from 3.2% to 13.6%. Extracts of three-year-old ginseng berry harvested in the 4th week of July exhibited significantly higher reducing power than other groups (Figure 2). Although the temporal changes of mean DPPH activity tended to be roughly similar to those of mean total phenols, the harvest time to exhibit the highest DPPH activity (4Y3W and 4Y4W) was different from that for total phenols (3Y4W). This discrepancy could be attributed to other antioxidant phytochemicals besides phenols in the ginseng berry extract, which warrants further investigation.

### 2.3. Elastase Inhibitory Activity of Ginseng Berry Extracts

**Figure 4:**
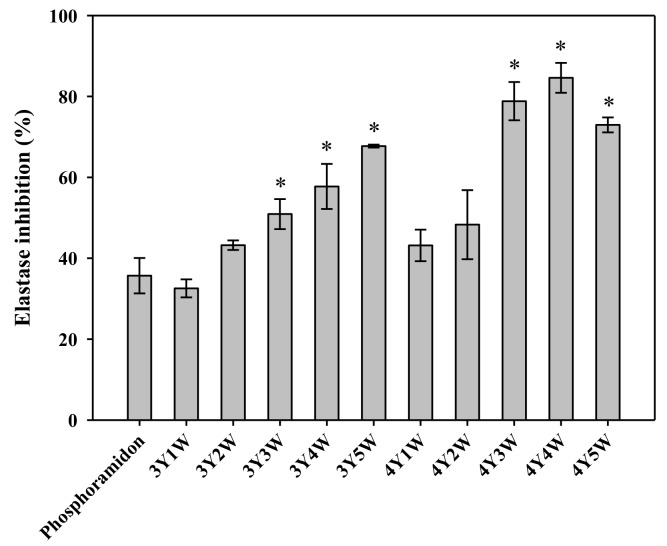
Elastase inhibitory activities of ginseng berry extracts harvested at various time points. Rectangular bars and their error bars represent means and standard deviations, respectively (*n* = 3). The ‘*m*Y*n*W’ on the x-axis means *m*-year-old ginseng berry harvested in the *n*th week of July. *, significantly higher than the ‘phosphoramidon’ group as positive control.

Figure 4 shows inhibitory effects of ginseng berry extracts on elastase activity. Elastase inhibitory activities of three-year-old ginseng berry extracts tended to increase from 32.5% to 70.0% as the harvest time was delayed from 1st week to 5th week of July. Those of four-year-old ginseng berry extracts showed a similar tendency, increasing from 43.2% to 84.6%. Extracts of three-year-old and four-year-old ginseng berry harvested in the 3rd, 4th, and 5th weeks of July exhibited significantly higher inhibitory activities than the phosphoramidon group (as positive control).

### 2.4. Contents of Ginsenosides in Ginseng Berry Extracts

**Figure 5:**
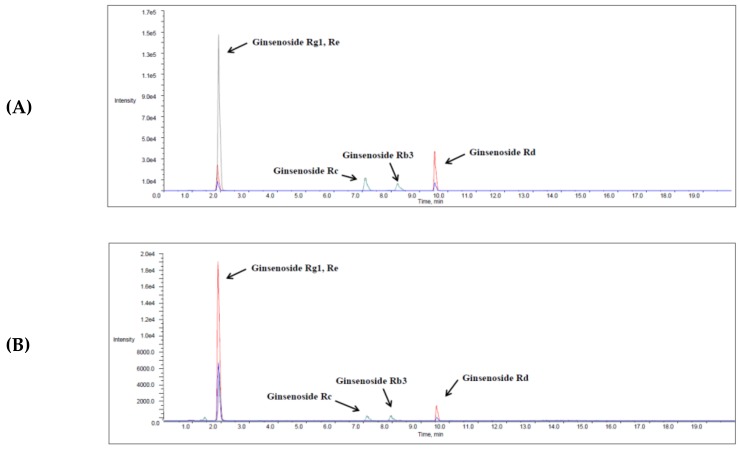

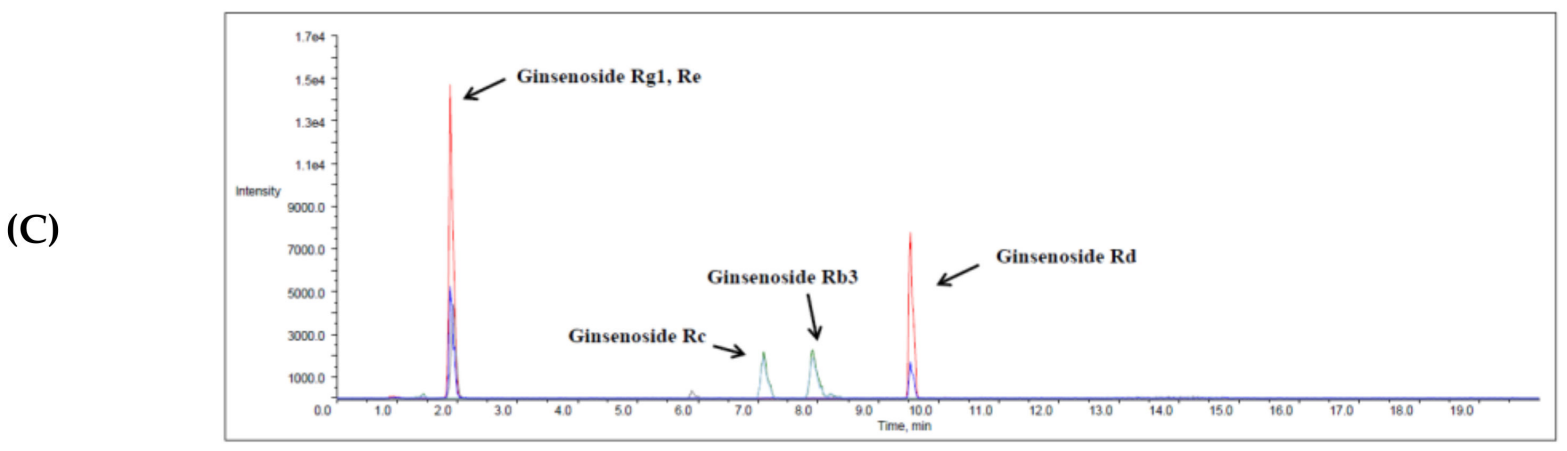
Representative chromatograms of ginsenosides Rb3, Rc, Rd, Re, and Rg1 in calibration standard (**A**), three-year-old ginseng berry extract sample (**B**), and four-year-old ginseng berry extract sample (**C**).

**Figure 6:**
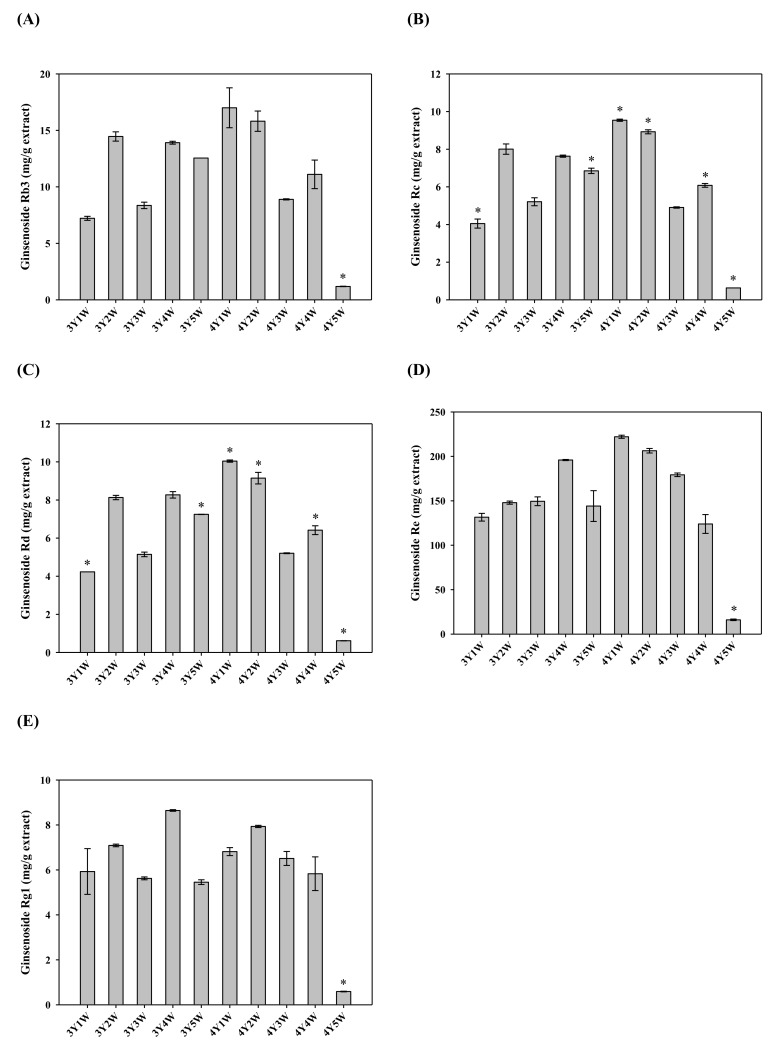
Contents of ginsenosides Rb3 (**A**), Rc (**B**), Rd (**C**), Re (**D**), and Rg1 (**E**) in ginseng berry extracts harvested at various time points. Rectangular bars and their error bars represent means and standard deviations, respectively (*n* = 3). The ‘*m*Y*n*W’ on the x-axis means *m*-year-old ginseng berry harvested in the *n*th week of July. *, significantly different from other groups.

Contents of ginsenosides Rb3, Rc, Rd, Re, and Rg1 in ginseng berry extracts were determined by LC-MS/MS analysis. Typical mass chromatograms are shown in Figure 5. Contents of five ginsenosides in extracts of ginseng berry harvested at various times are shown in Figure 6. As shown in Figure 6B,C, Rc and Rd contents were significantly higher in extracts of four-year-old ginseng berry harvested in the 1st week of July than those in other groups. Similarly, Rb3 and Re contents tended to be the highest in extracts of four-year-old ginseng berry harvested in early July (Figure 6A,D). However, Rg1 content exhibited a slightly different tendency from other ginsenosides. It tended to be the highest in extracts of three-year-old ginseng berry harvested in the 4th week of July and four-year-old ginseng berry harvested in the 2nd week of July (Figure 6E). Contents of all ginsenosides studied were the lowest in extracts of four-year-old ginseng berry harvested in the last week of July.

### 2.5. Effects of Harvest Time on Chemical Constituents and Biological Activities of Ginseng Berry Extracts

In both 3- and four-year-old ginseng berry extracts, antioxidant (DPPH radical scavenging activity and reducing power) and anti-elastase activities tended to increase as berries ripened from the first week to the last week of July. However, contents of ginsenosides except Rg1 tended to be higher in four-year-old ginseng berries harvested in early July than those in other groups. These results indicate that biological activities and ginsenoside profiles of ginseng berry extracts depend on their age and harvest time in July, suggesting a need to optimize harvest time for the development of functional foods and medicinal products containing ginseng berry extracts. To the best of our knowledge, this is the first study to report the impact of harvest time on antioxidant and anti-elastase activities as well as ginsenoside contents of ginseng berry extracts.

## 3. Materials and Methods

### 3.1. Plant Materials

Ginseng berry was harvested from three-year-old and four-year-old ginseng cultivated in a local farm (Healthy Sam-Farm, Jeonbuk, Korea) every week from July 1 to July 30, 2017. Dried ginseng berry of 25 g was extracted with 70% ethanol at room temperature for 72 h. After removing ethanol, residual water part was freeze-dried and then stored at −70 °C before analysis.

### 3.2. DPPH Free Radical Assay

Antioxidant activity was determined with 2,2-diphenyl-1-picrylhydrazyl (DPPH) radical scavenging assay. Briefly, 1 mL sample solution (final concentration: 1–20 mg/mL; dissolved in DDW) was added to 0.4 mM DPPH sample solution (1 mL; dissolved in methanol) and then vortex-mixed. The resultant mixture was allowed to react at room temperature in the dark for 10 min. Its absorbance at 517 nm was then measured using a microplate reader (Perkin Elmer, Waltham, MA, USA). DPPH free radical scavenging activities of samples in terms of their IC_50_ (μg/mL) values were evaluated. Vitamin C was used as a positive control.

### 3.3. Reducing Power

Reducing power was determined using a modified reducing power assay. Briefly, sample (0.1 mL) was added to 0.2 M sodium phosphate buffer (0.5 mL) and 1% potassium ferricyanide (0.5 mL), followed by incubation at 50 °C for 20 min. Subsequently, 10% trichloroacetic acid solution (0.5 mL) was added to the reaction mixture followed by centrifugation at 12,000× *g* for 10 min. The supernatant was mixed with distilled water (0.5 mL) and 0.1% iron (III) chloride solution (0.1 mL). The absorbance of the resulting solution was measured at 700 nm. Reducing powers of samples are expressed as vitamin C equivalents [21].

### 3.4. Determination of Total Phenolic Content

Total phenolic content was determined by Folin–Ciocalteu assay. Briefly, 1 mL sample (final concentration: 5 mg/mL) was mixed with 1 mL of 2% sodium carbonate solution and 1 mL of 10% Folin–Ciocalteu’s phenol reagent. After incubating the mixture at room temperature for 10 min, its absorbance was measured at 750 nm using microplate reader and compared with the calibration curve of gallic acid. Data are expressed as milligrams of gallic acid equivalents per gram of sample [21].

### 3.5. Determination of Elastase Inhibitory Activity

Elastase inhibitory activity was determined as previously described [22]. Briefly, 10 μL elastase derived from porcine pancreas (10 μg/mL) was mixed with 90 μL of 0.2 M Tris-HCl, 100 μL of STANA (2.5 mM, *N*-Succinyl-Ala-Ala-Ala-*p*-nitroanilide), and 50 μL of the sample and incubated at 37 °C for 30 min. The reaction mixture was then centrifuged at 15,000× *g* for 10 min to obtain supernatant. The absorbance of the supernatant was measured at 405 nm using a microplate reader. Phosphoramidon, an inhibitor of elastase from *Pseudomonas aeruginosa*, was used as a positive control.

### 3.6. Determination of Ginsenoside Contents

Contents of ginsenosides Rb3, Rc, Rd, Re, and Rg1 were determined by high-performance liquid chromatography-tandem mass spectrometry (LC-MS/MS) analysis. The LC-MS/MS system consisted of a Sciex HPLC system coupled with a triple quadrupole mass spectrometer (Triple Quad 4500, AB Sciex, Framingham, MA, USA). The mobile phase for the HPLC system consisted of water containing 0.1% formic acid (solvent A) and acetonitrile containing 0.1% formic acid (solvent B). It was eluted at 0.4 mL/min. A gradient elution protocol was used: solvent A:solvent B, *v/v* ramped from 72:28 to 65:35 for 6 min; ramped from 65:35 to 0:100 for 4 min; held at 0:100 for 1 min; back to 72:28 for 4 min; and then held at 72:28 form 5 min. Chromatographic separation was performed using a reversed-phase column ZORBAX Eclipse Plus (C18, 3 × 100 mm, particle size 1.8 μm; Agilent, Santa Clara, CA, USA), which was maintained at 40 °C. To avoid contamination by particles, the mobile phase was filtered through a 0.45 μm filter device (PEEK, Supelco, Taufkirchen, Germany) before use. The mass spectrometer was operated in the positive ion mode using multiple reaction monitoring (MRM). The following ion source parameters were used: temperature, 600 °C; collision gas pressure, 9 mTorr; sheath gas pressure, 40 Arb; and auxiliary valve flow rate, 10 Arb. Detailed mass spectrometry parameters are listed in Table 3.

### 3.7. Statistical Analysis

A *p*-value < 0.05 was considered statistically significant using *t*-test for comparing unpaired two means or analysis of variance (ANOVA) with post-hoc Tukey’s HSD test for comparing unpaired three means. All data are rounded to three significant digits and expressed as mean ± standard deviation.

## 4. Conclusions

The present study demonstrated that antioxidant and anti-elastase activities tended to increase as berries ripened from the first week to the last week of July in both three- and four-year-old ginseng berry extracts, and the contents of ginsenosides except Rg1 tended to be the highest in four-year-old ginseng berries harvested in early July. These findings indicate that biological activities and ginsenoside profiles of ginseng berry extracts are dependent on their age and harvest time in July, suggesting the importance of harvest time in developing functional foods and medicinal products of ginseng berry extracts. To the best of our knowledge, this is the first report on the influence of harvest time on biological activity and ginsenoside contents of ginseng berry extracts.

## Figures and Tables

**Table 1:** Chemical constituents and pharmacological activities of ginseng berry extracts reported in previous literature.

Ext. Solvent	Constituent	Activity	Region	Effective Dose (mg/kg) (route/animal/day)	Estimated Human Dose (mg/60 kg/day)	Ref.
Ethanol Water	Rb1, Rb2, Rd,Re,Rf, Rg1, Rg2, 20SRg3, Rg6, Rh1, Rh4,Rk1,Rk3, F1,F4	Hepatoprotective	South Korea	100–500 (PO/rat)	972.4–4862	[3]
Ethanol	Polysaccharide K	Anti-immunosenescent		30 (PO/mouse)	146	[4]
Butanol	Re	Antidiabetic	China	150 as ext.5–20 as Re(PO/mouse)	729 as ext.24.3–97.3 as Re	[10]
ND	Polysaccharides	Antidiabetic	USA	150 (IP/mouse)		[11]
Ethylacetate	Re	Antidiabetic	South Korea	20–50 (PO/mouse)	97.3–243.3	[12]
70% ethanol	Rb1, Rb2, Rc, Rd, Re, Rg1, Rg2	Penile erection	South Korea	20–150 (PO/rat)	194.5–1458.7	[13]
70% ethanol		Antipigmentation		In vitro		[14]
Butanol	Rg1, Re, Rh1, Rg2, Rb1, Rc, Rb2, Rb3, Rd, Rg3, 20R-Rg3, Rh2	Anticancer	USA	50 (PO/mouse)	243.3	[15]
Water	Rb1, Rb2, Rc, Rd, Re, Rf	Blood circulation		50–150 (PO/rat)	486.2–1458.7	[16]

**Table 2:** Drying and extraction yields of ginseng berry extracts.

Sample	Drying (%, *w/w*)	Extraction (%, *w/w*)
3Y1W	29.7	11.2
3Y2W	31.2	11.0
3Y3W	34.8	8.8
3Y4W	32.2	11.9
3Y5W	31.3	11.4
4Y1W	32.0	10.4
4Y2W	33.1	10.8
4Y3W	32.8	11.2
4Y4W	30.7	9.2
4Y5W	29.9	12.6

**Table 3:** Mass spectrometry parameters for the detection of ginsenosides.

Compound	Q1 mass	Q3 mass	Collision Energy (V)
Rb3	969.7	789.7	46
Rc	1101.7	335.0	65
Rd	969.8	789.4	60
Re	1101.7	335.0	65
Rg1	823.5	643.5	50
